# Electrospinning Fabrication Methods to Incorporate Laminin in Polycaprolactone for Kidney Tissue Engineering

**DOI:** 10.1007/s13770-021-00398-1

**Published:** 2021-10-29

**Authors:** Büsra Baskapan, Anthony Callanan

**Affiliations:** Institute for Bioengineering, School of Engineering, University of Edinburgh, Faraday Building, King’s Buildings, Colin Maclaurin Road, Edinburg, EH9 3DW UK

**Keywords:** Laminin, Kidney, Emulsion, Scaffold, Tissue engineering

## Abstract

*****BACKGROUND***:**

Today’s treatment options for renal diseases fall behind the need, as the number of patients has increased considerably over the last few decades. Tissue engineering (TE) is one avenue which may provide a new approach for renal disease treatment. This involves creating a niche where seeded cells can function in an intended way. One approach to TE is combining natural extracellular matrix proteins with synthetic polymers, which has been shown to have many positives, yet a little is understood in kidney. Herein, we investigate the incorporation of laminin into polycaprolactone electrospun scaffolds.

*****METHOD***:**

The scaffolds were enriched with laminin via either direct blending with polymer solution or in a form of emulsion with a surfactant. Renal epithelial cells (RC-124) were cultured on scaffolds up to 21 days.

*****RESULTS***:**

Mechanical characterization demonstrated that the addition of the protein changed Young’s modulus of polymeric fibres. Cell viability and DNA quantification tests revealed the capability of the scaffolds to maintain cell survival up to 3 weeks in culture. Gene expression analysis indicated healthy cells via three key markers.

*****CONCLUSION***:**

Our results show the importance of hybrid scaffolds for kidney tissue engineering.

**Supplementary Information:**

The online version contains supplementary material available at 10.1007/s13770-021-00398-1.

## Introduction

According to the World Health Organization 10% of the world’s population suffer from acute or chronic kidney diseases [1]. Given that the number of lives lost from renal failure has increased 32% from 2005 to 2015 [2], it is a huge global burden for society. Limitations of current treatment options, such as palliative nature of dialysis and the shortage of available donor organs for transplantation are just some of the factors contributing to the increase. Alongside this, drug therapy given to patients has been shown to lead to drug–induced toxicity, and is stated as being responsible for 19–25% of all cases of severe renal failure [3]. This paucity of current treatment options for renal failure reveals the need for more reliable and effective methods.

To better understand disease progression and find a way to enhance today’s treatment options, tissue engineering has been highlighted as a potential way forward [4, 5]. One of the endeavours in this field is creating a three-dimensional foundation that can provide a suitable niche for cells to behave as they would in vivo. For this purpose, electrospinning of polymers has been widely used to produce fibrous scaffolds as it enables easy manipulation of chemical structure and fibre architecture [6–8]. Nevertheless, scaffolds from synthetic polymers lack natural biomolecular signals which cells benefit from to modulate their functions. These signals can be provided by either adding bioactive agents like proteins into the polymer through blending or coating to recapitulate natural tissue [9–11].

Recently evidence has shown the advantages of protein addition into polymeric scaffolds, with the desired outcome and response due in part to its composition, binding method and presentation [12, 13]. However, a major challenge is to preserve the bioactivity, which is important in maintaining cell attachment and behaviour [14, 15]. Also additional characteristics can be inadvertently effected by washing and sterilization steps which could cause damage or removal [16, 17]. Alternatively, controlled inclusion of protein into polymer fibres can lead to increased stabilisation and function. While uncontrolled addition can allow large fluctuations in release kinetics which can have advantages and disadvantages [14, 18]. Furthermore encapsulation can allow controlled release and protection [19].

Although stimulation of cells is achievable by adding biomolecules in polymeric scaffolds [9, 12, 13], to date little has been achieved in kidney tissue engineering. Laminin is one of the major component of kidney basement membrane that plays an important role in development and cell attachment [20]. The protein has been shown to provide stimulation in several tissues but its effect in kidney tissue engineering is not fully investigated [21–23]. Therefore, we present polymeric scaffolds combined with laminin via different electrospinning approach investigating renal cell response.

## Materials and method

### Scaffold fabrication

**Fig. 1:**
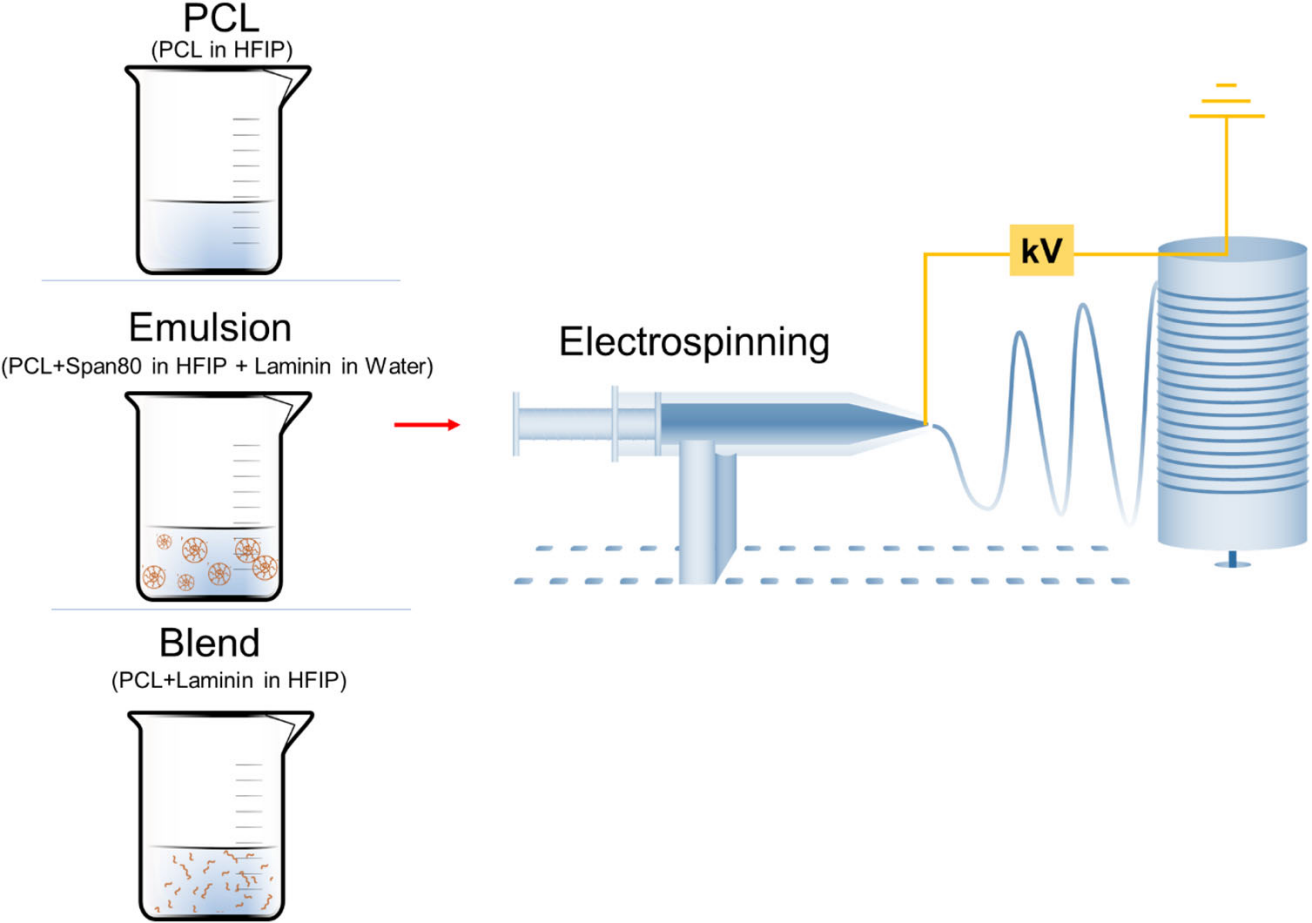
Schematic representation of scaffold fabrication

Scaffolds were produced using electrospinning platform (EC-DIG, IME Technologies, Netherlands) (Fig. 1). 14% (w/v) polycaprolactone (PCL) (M_n_ = 80 kDa) (Sigma-Aldrich, Gillingham, UK) was dissolved in 1,1,1,3,3,3-hexafluoro-2-isopropanol (HFIP) (Manchester Organics, UK) and left agitation on a roller mixer until electrospinning through a syringe connected to 0.8 mm needle at a flow rate of 1 ml/h. Fibres were collected on an aluminium foil covered rotating mandrel with 250 rpm from 22 cm distance and via voltage of 14 kV. For blend solution, laminin (Santa Cruz Biotechnology, Heidelberg, Germany) was directly added to PCL solution, mixed and electrospun under 12.1 kV voltage using 0.8 mm needle at a flow rate of 1.3 ml/h on a 250 rpm rotating mandrel from 17 cm distance. Emulsion solution was prepared by mixing 30% (w/v) aqueous phase of laminin dissolved in deionized water with oil phase of PCL solution consisting of 0.4% Span80 (v/v) (Sigma-Aldrich) at 1:60 (v/v) ratio. The spinning solution was then fed at 1.2 ml/h flow rate to a 0.8 mm needle and collected on a mandrel rotating with 250 rpm under 12.9 kV voltage with a 17 cm distance from needle to mandrel.

### Scaffold characterization

#### Mechanical testing

Mechanical properties of the scaffolds were measured based on previous studies of our group [24]. 20 mm in gauge length and 5 mm in width rectangular samples were used to measure tensile properties of scaffolds in an Instron 3367 machine (Instron, High Wycombe, UK). 10% strain per minute was set to load samples uniaxially. From stress–strain curves ultimate tensile strength and Young’s modulus between 0 and 10% strain in 2% intervals were characterized from average of independent replicates.

#### Morphology of scaffolds

Images of scaffolds were taken by using scanning electron microscope (SEM) (Hitachi TM4000 Plus) under 15 kV voltage from a 10 mm distance after coated with gold by an Emscope FLM-007 sputter coater. Fibre diameter of scaffolds was measured by Diameter J, using three different SEM images for each scaffold group.

### Cell culture

A human kidney epithelial cell line (RC-124) (CLS, Germany) was used to seeding scaffold. Cells in 15th passage were suspended in McCoy’s 5A media containing L-Glutamine supplemented with 1% antibiotic–antimycotic and 10% foetal bovine serum (all media and supplements from Gibco, ThermoFisher Scientific, Loughborough, UK). Cells in T75 flasks were incubated in 5% CO_2_ at 37 °C. Accutase was used as dissociation agent to detach cells.

Scaffolds were treated with 70% ethanol for 30 min and PBS three times with 15 min intervals to sterilize and later soaked into the media overnight prior to seeding. 12 mm diameter scaffolds were first seeded with 50 μl of cell suspension containing 63 000 cells for each scaffold and then an additional 400 μl of media was added after 1 h of incubation. Scaffolds were cultured for 1, 7, 14 and 21 days before analyses, with media changed 3 times a week.

### Morphology of cells on scaffolds

Osmium staining was carried out to visualize seeded scaffolds at day 1, 7 and 14. Scaffolds (*n* = 2) were fixed in 4% (v/v) glutaraldehyde overnight and stored in PBS at 4 °C until incubated in 0.1% (v/v) osmium for 30 min, followed by washing with distilled water four times. Samples were then dehydrated in increasing concentrations of ethanol from 30 to 100% (v/v) with 30 s intervals. Scaffolds were then placed in HDMS for 1 min. HDMS was then replaced and left to evaporate overnight in a fume hood. After staining all images of seeded scaffolds were taken by SEM after coated with gold by sputter coater.

### Cell viability

CellTitre-Blue® assay (Promega, Southampton, UK) was used to determine cell viability according to manufacturer’s instruction on the day of each time point. Cell seeded scaffolds were washed 3 times in PBS and then a mixture of CellTitre-Blue® assay with cell culture media (1:5) was added on scaffolds to incubate for 3.5 h. Fluorescence was read using a microplate reader (Modulus II 9300–062, Turner Biosystems) at Ex 520 nm Em 580–640 nm.

### DNA quantification

For total DNA content samples were first freeze dried overnight and then incubated in a papain digest solution containing 2.5 units ml^−1^ papain, 5 mM cysteine HCL and 5 mM EDTA in PBS (all reagents from Sigma-Aldrich) at 65 °C for 48 h and periodically vortexed until used. Total DNA content of the samples was calculated using a Quant-iT^TM^PicoGreen® assay kit (ThermoFisher) as per the manufacturers’ instructions. Fluorescence values were read using a microplate reader at Ex 490 nm Em510–570 nm.

### Gene expression (RT-qPCR)

Reverse transcription quantitative real-time PCR (RT-qPCR) was started with RNA extraction from scaffolds and cells homogenized in Tri-Reagent^®^ (Invitrogen, ThermoFisher). RNA was isolated from DNA and proteins with the addition of chloroform and ethanol, respectively and harvested using an RNeasy kit (Qiagen, Manchester, UK) as per manufacturer’s instructions. The cDNA from RNA using an InProm-II kit (Promega) according to manufacturer’s instructions was produced and measured in PCR machine (Applied Biosystems Proflex PCR system). Last step was carried out by SensiFAST™ SYBR^®^ Hi-ROX kit (Bioline, London, UK) according to manufacturer’s protocol. Three genes were used for this study; Epithelial cadherin (E-CAD), Alanyl aminopeptidase (ANPEP) and Kidney injury molecule-1 (KIM-1) and their primers [6] (Sigma-Aldrich) were given in Supplementary material. Measurement was taken using LightCycler 480 Instrument II (Roche). The gene expression levels were normalised to a housekeeping gene (Glyceraldehyde-3-phosphate dehydrogenase-GAPDH) and RC-124 cells on tissue culture plastic and calculated with 2^−ΔΔCt^ method [25].

### Statistics

All statistics were performed using Minitab software. A one-way analysis of variance (ANOVA) and Tukey post hoc test were chosen to assess changes with 95% confidence interval. Significance was considered with a *p*-value < 0.05. Data is presented with standard deviation (SD).

## Results

### Scaffold characterization

**Fig. 2:**
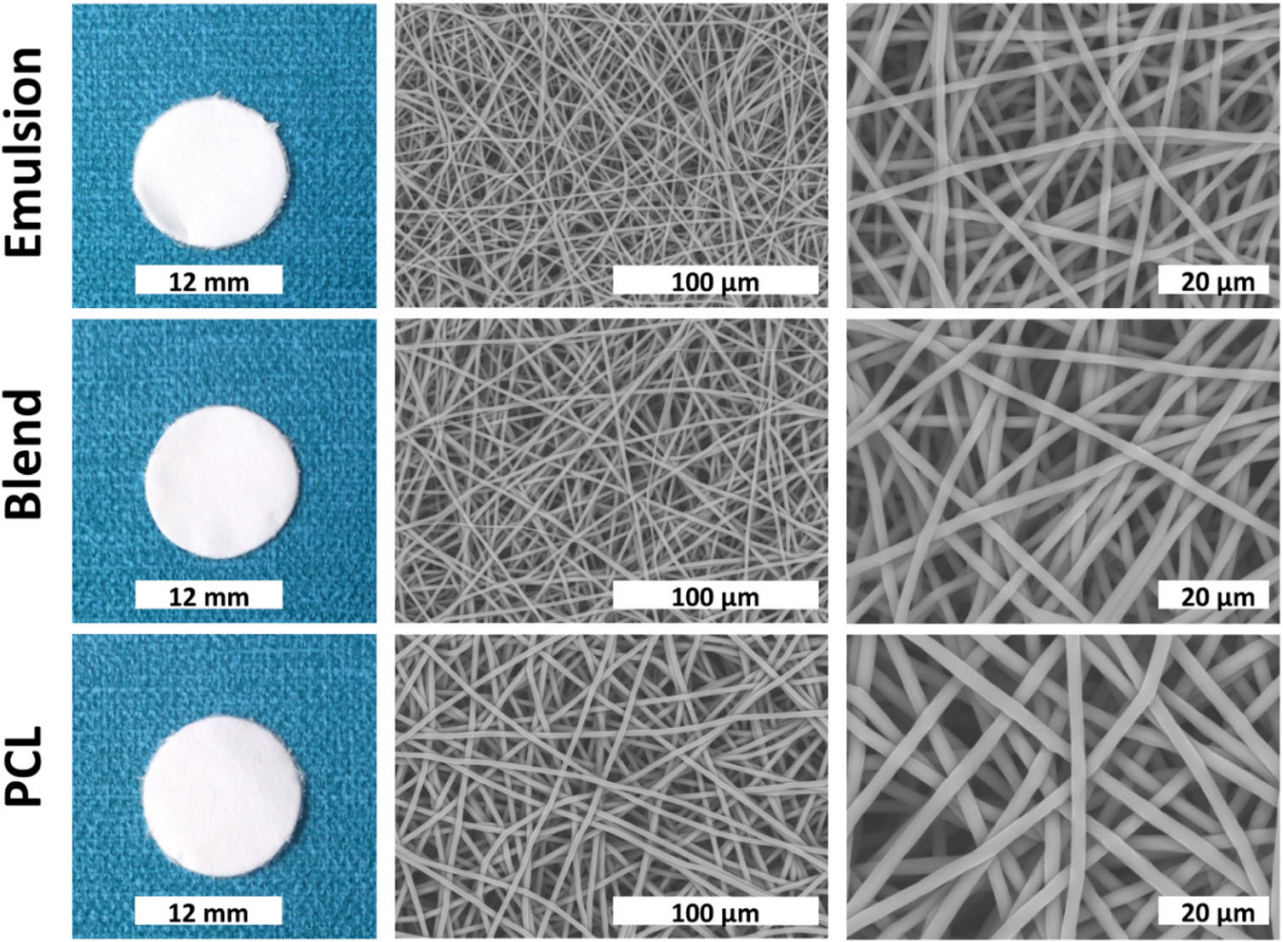
Gross and SEM images of scaffolds at different magnification (× 1500 and × 4000 respectively)

SEM and gross images (Fig. 2) show similar architecture of scaffolds that were successfully fabricated by electrospinning. Average fibre diameter of scaffolds ranges from 1.81 ± 0.24 to 2.83 ± 0.21 µm (Table 1) where emulsion group has the smallest. Despite the slight decrease in size fairly uniform orientation within scaffolds was obtained.

**Table 1:** Fibre diameter and tensile strength of the scaffolds

Mechanical properties	PCL	Blend	Emulsion
Fibre diameter (µm)	2.83 ± 0.21	2.16 ± 0.57	1.81 ± 0.24
Ultimate tensile strength (kPa)	11.33 ± 2.97	21.83 ± 1.82	18.06 ± 2.65

*n* = 3, ±  = SD

**Fig. 3:**
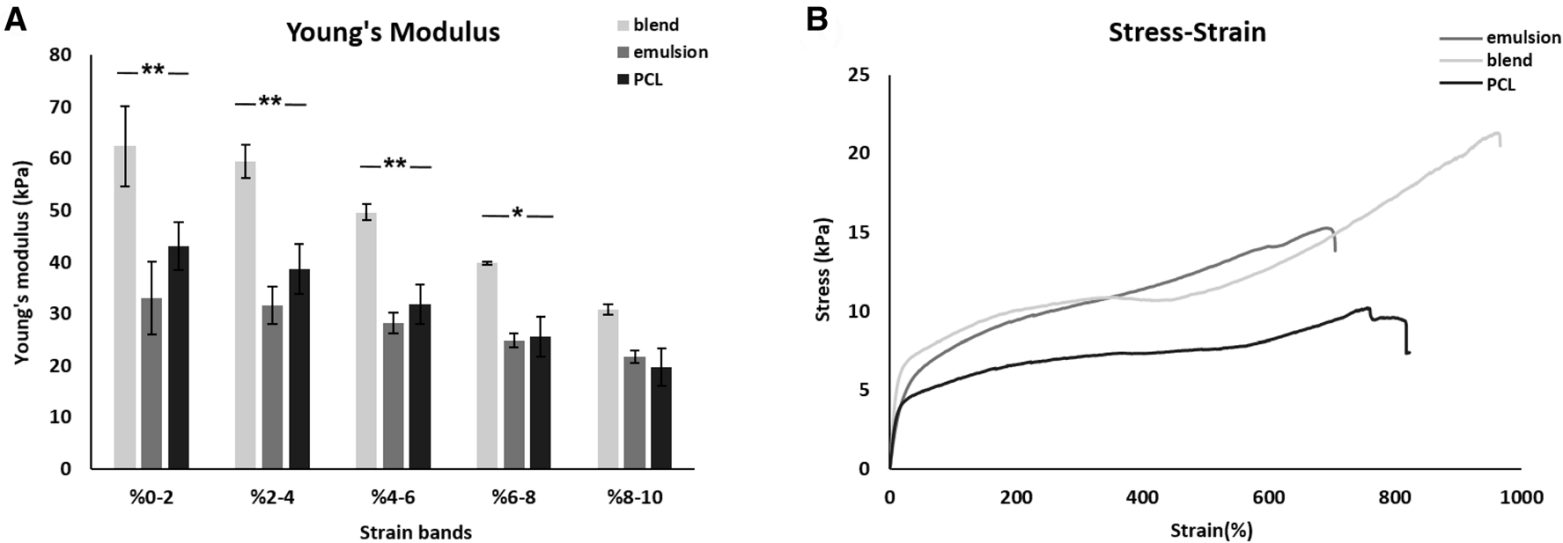
Young’s modulus **A** and Stress–Strain **B** graphs of scaffolds before cell seeding. Error bars = SD, *n* =  > 3. *p < 0.01; **p < 0.001; one-way ANOVA

Laminin has changed the mechanical properties of scaffolds as shown in Table 1 and Fig. 3. Ultimate tensile strength increased from 11.33 to 21.83 kPa with protein addition while difference between blend and emulsion groups is not significant. Average Young’s modulus for the blend was found significantly higher up to 8% strain than others. While emulsion scaffold was lower than PCL alone. Elastic modulus values for each scaffold dropped over stretching 20% where all started to become plastic. As seen in stress–strain graph incorporation of laminin made polymeric scaffold more elastic.

### Cell attachment and survival

**Fig. 4:**
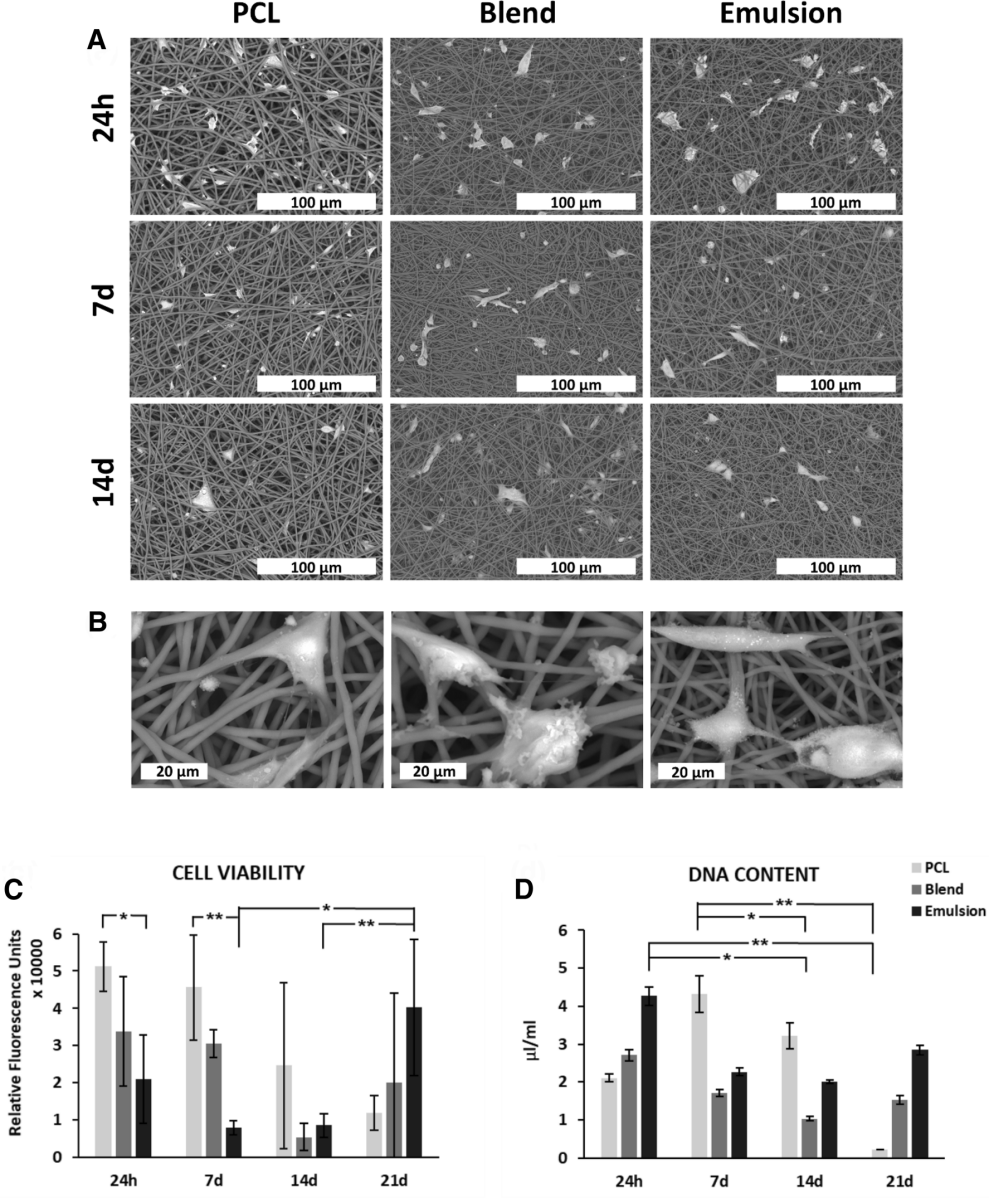
**A** SEM images of RC-124 cells on scaffolds (× 800). **B** Enlarged images of cells on scaffolds showing cell–cell and cell-fiber interaction (× 4000). **C** Cell viability of 24 h, 7d, 14d and 21d of cell culture on scaffolds using CellTitre Blue assay. **D** DNA content of cell seeded scaffolds in different time points of culturing (Picogreen DNA quantity assay). Values normalised to scaffolds-free control. Error bars = SD, *n* = 5. *p < 0.05; **p < 0.01; one-way ANOVA

SEM images of cell seeded scaffolds exhibit that kidney cells can keep attached to the fibres up to 14 days (Fig. 4A) and penetrate into the pores. While the number of cells on scaffolds seems lowering over subsequent time point, shape of cells changed into more elongated especially on blend and emulsion groups. Likewise, magnified images (Fig. 4B) revealed the cell-cell and cell-fibre interaction.

Cell viability results indicate cell survival up to 3 weeks as the test is based on cell metabolism. This was also supported by SEM images of cell seeded scaffolds. Interestingly, cell viability is higher in PCL than laminin incorporated scaffolds (in particular emulsion) up to 7 days (*p* < 0.05) (Fig. 4C). Blend group is also higher than emulsion, but not significant. This trend changed in favour of emulsion after two weeks (*p* < 0.001).

Significant differences in DNA content of the scaffolds were found as seen in Fig. 4D. There was a decrease in DNA content for PCL scaffold between 7 and 21 days (*p* < 0.01). Additionally, emulsion at day 1 was significantly higher than blend at 14 days and PCL at 21 days (*p* < 0.05).

### Gene expression

**Fig. 5:**
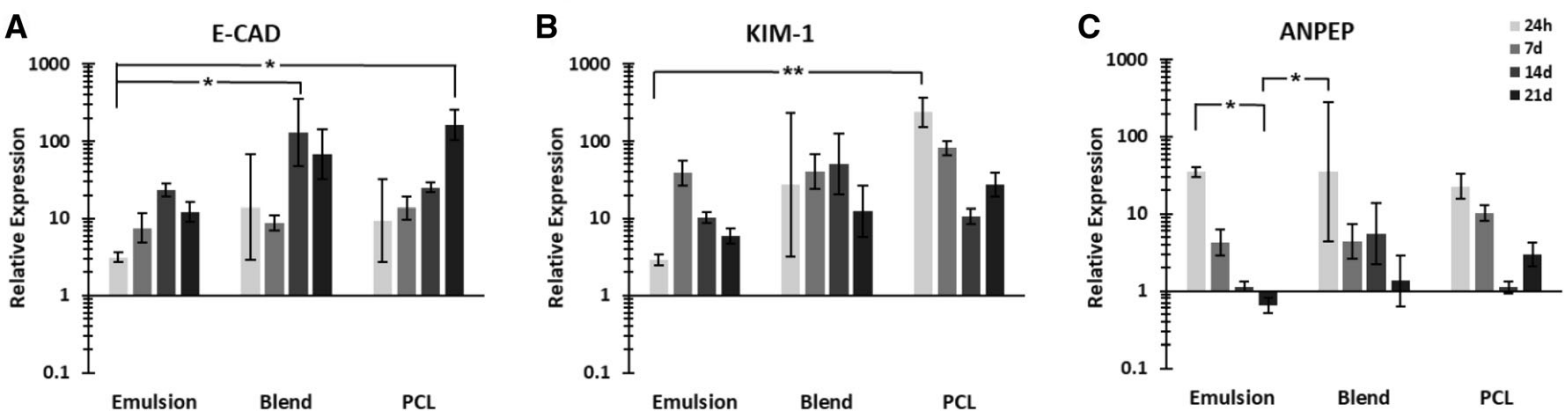
RC-124 (kidney primary epithelial cells) gene expression of E-CAD **A** KIM-1 **B** and ANPEP **C** on emulsion, blend and PCL scaffolds in 24 h, 7d, 14d and 21d. Results normalised to GAPDH. Error bars = SD, *n* = 5. *p < 0.05; **p < 0.01; one-way ANOVA. E-CAD: E-cadherin, KIM-1: Kidney injury molecule-1, ANPEP: Alanyl aminopeptidase, GAPDH: Glyceraldehyde-3-phosphate dehydrogenase

RT-qPCR results show the trends for three key genes (Fig. 5). Significant decrease over time in relative expression of ANPEP in the emulsion scaffolds was noted. Blend and PCL scaffolds had also a general decrease over three weeks, but this was not significant. E-CAD gene expression of cells on all scaffolds was upregulated over time but only significance was found between the emulsion at 24 h, the blend at 14 days and PCL at 21 days. KIM-1 gene had lower expression in emulsion group than PCL at same time-point, along with the downregulation between first and third week for the same group.

## Discussion

Combination of natural protein with synthetic polymers has recently gained an interest in tissue engineering since it allows taking advantages of each material to create a hybrid scaffold [26–29]. One important advantage is to overcome lack of biological signals in polymers to induce seeded cells towards the desired outcome [30, 31]. Still, retaining bioactivity of protein remains a hurdle as during processing and fabrication it can be exposed to conditions that might destroy its structural integrity [32]. Herein, we investigated hybrid scaffolds that incorporate PCL with laminin for kidney tissue engineering. Laminin concentration has been reported an important factor on both cell attachment and functionality [20, 33]. Concentrations present in literature for electrospinning of laminin in polymer solution vary from 4 to 30 µg/ml [21, 22, 34, 35]. These studies showed cell viability and proliferation over time, our selected concentration falls within this band at 5 µg/ml. This concentration further justified by corresponding to Kapadia’s finding of minimal binding of porcine kidney cells to laminin coated surface [20].

Laminin is a vital component of the kidney basement membrane, mediating cell attachment [20]. It also plays a key role for other ECM proteins to bind to cell surface antigens [36]. One of the most used methods to combine laminin with PCL in the literature is coating [33, 37]. This is accompanied by many stability issues with a major issue of coating removal during the sterilization steps [12, 16]. Alternatively, electrospinning of protein in polymer solution is an option for stable incorporation and release kinetics [18, 22]. The main concern in this technique is that solvent can denature protein composition [38, 39]. Consedering these problems we have chosen two methods in this study, blend and emulsion electrospinning. Emulsion systems have been reported to protect protein from solvents, this is in part due to a water carrying protein which is covered with surfactant [40, 41]. During electrospinning emulsions are forced to unify due to electrostatic repulsion and thus can produce core–shell fibres [42]. In the blend systems protein can be distributed throughout the fibre due to direct addition into polymer solution. This localisation of protein in fibre can affect the release profile and accordingly the cell response [13, 19]. These factors rationalise the need to investigate a number of laminin incorporation methodologies as outlined in this study.

We utilized electrospinning to produce scaffolds as it is a good and repeatable technique to manufacture porous structure, where fibres can be altered by changing spinning parameters. We were able to spin hybrid scaffolds with a similar morphology (Fig. 2). This consistency enables us to eliminate the size effect on cell behaviour [43–45] and thus ascribe the results in part to the laminin. The range between three groups was able to be tailored with the maximum variation of 1 µm. Some studies reported similar findings that addition of biomolecules results in thinner fibres [14, 23].

We noted interesting results in mechanical properties due to laminin incorporation method. Inclusion of laminin affected the elasticity of the blend group but not the emulsion. Young’s modulus of the blend is highest, while the difference at higher strains becomes smaller. Hybrid scaffolds have a higher ultimate tensile strength compared to PCL. This impact has been demonstrated with other proteins and polymers [34, 46]. An increase in Young’s modulus have been linked to positive effects such as cell proliferation and spreading, but also negative responses in particular apoptosis [47, 48]. This fundamental also applies to decreased stiffness where studies have shown that there can be a positive influence such as increased focal adhesion properties and also some negatives with decreased cell motility [49, 50]. When considering the native kidney biomechanics, the environment has vast array of mechanical properties, this varies from single fibrils to bulk ECM (3 Pa to 7.5 GPa) [51, 52] While recapitulating these properties is difficult within a scaffold platform, the electrospinning technique can capture some of the fundamentals such as the fibrous architecture and mechanical properties (23 kPa to 3.8 MPa) which can fall within the range of native tissue [53, 54].

Aspects of laminin addition on renal cells were investigated with viability and DNA quantification. Results point out that the scaffolds are capable of maintaining cell survival and modulate their response over time. Although the viability on PCL was significantly higher up to 1 week, this trend changed at week 3 in favour of emulsion group which could be from laminin release or presentation. Yu’s group reported similar observation for cardiac tissue engineering [12]. Likewise this reasoning could be applied to our results, but it requires further investigation. Results also showed that the hybrid scaffolds are suitable base to create environment for kidney cells. This was supported by SEM images (Fig. 4A) where cell-cell and cell-fibre interaction can be seen clearly in enlarged images (Fig. 4B). The nature of these interactions can be studied by immunostaining in future works. These current findings are very promising sign for an in vivo-like niche.

Gene expression revealed the impact of laminin. E-CAD, a key marker for formation of cell junctions in epithelial tissues [55], increased over time in all groups. To further corroborate E-CAD activity SEM images in Fig. 4B exhibit cell binding to each other, a fundamental driver of expression. Increased expression has been previously linked to improved cell growth and barrier function on substrates consisting of laminin [56, 57]. Interestingly, upregulation can be an indication of monolayer formation, an important aspect for function [55]. ANPEP is another gene found in renal membrane, associated with multiple signalling pathways from adhesion, angiogenesis and proinflamatory mediators [58]. Our result showed a downward trend in expression on all scaffolds with a statistically significance in the emulsion group. As high expression has been found in many cancer studies [59] downregulation over time can possibly indicate a positive sign while cells are adapting to environmental changes. KIM-1 is a novel marker related to acute kidney injury, epithelial damage and stress [60]. In our study expression of this gene in the emulsion was considerably lower than PCL in 24 h, consistent with a recent study showing downregulation by laminin inclusion [61]. It can be hypothesized that a decrease in expression may be an indication of a decrease in cell stress. The total of these findings from these three key markers suggest that hybrid scaffolds support renal cells and provide a suitable environment over time.

Change in cell behaviour with laminin incorporation into polymeric scaffold has previously been linked to the activation of certain molecular mechanisms. One mechanism is that cell attachment via pro-adhesive laminin sequences such as RGD, IKVAV and YIGSR can provide binding sites to cells [12, 32]. This is a commonly mentioned mechanism by researchers who have reported increased cell attachment on scaffolds with laminin [21–23]. Another mechanism is the modified hydrophilicity by the protein addition, which can lead to an increased cellular interaction, this has been shown in respect to laminin-mixed scaffold compared to PCL alone [32, 62]. A third mechanism could be a change in surface stiffness due to laminin incorporation, which is linked to cell behavioural changes in migration, differentiation and proliferation [34]. Interestingly we noted that our hybrid scaffolds had an increased elasticity and notable difference in attachment. While other mechanisms exist, it is likely that it is a combination of many of these and not any specific one.

Despite these promising results there is still some future considerations for this work. The first is the damage effects that solvent may cause on the protein composition. As HFIP can denature several proteins this is a major concern for blended scaffolds [38, 63]. In emulsion scaffolds we believe it is not the case since surfactant offers a form of protection. However, further investigation is crucial to see if concentration of surfactant is sufficient to prevent such a large protein from interaction with the solvent. Secondly, voltage can alter protein structure [64] yet it has been reported that it may not have an adverse effect on laminin, but it is worthy to note that they did not look at the effect of voltage in these studies [13, 35]. Also our results, in particular significant differences between blend and PCL groups, can be attributed to a retained bioactivity. Despite these limitations we were able to showcase the effects of laminin inclusion in polymeric scaffolds.

This study has revealed successful electrospinning of laminin in PCL scaffolds and their support for renal cells. Protein inclusion changed the mechanical properties of pure polymer to a more elastic structure. Renal cells on the scaffolds were metabolically active, which points out to biocompatibility of scaffolds. Additionally, change in expression levels of three important genes shows healthy function of cells on the scaffolds. We believe that the total of these results highlight the need for further investigation on combinations of ECM proteins with synthetic polymers in kidney tissue engineering applications.

## Supplementary Information

Below is the link to the electronic supplementary material.

Supplementary file1 (DOCX 19 kb)
