# Supplementary material for: Electrospinning Fabrication Methods to Incorporate Laminin in Polycaprolactone for Kidney Tissue Engineering
Source: Tissue Eng Regen Med. 2021 Oct 29;19(1):73–82. doi: 10.1007/s13770-021-00398-1 (PMC8782962; doi:10.1007/s13770-021-00398-1)
Supplement: Supplementary file 1 — Supplementary file1 (DOCX 19 kb) [file 13770_2021_398_MOESM1_ESM.docx]

***Table S1****. Primer sequences for the genes used in PCR.*

| **Name** | **Acronym** | **Primers** |
| --- | --- | --- |
| Epithelial cadherin | E-CAD | Forward: 5′-AGCGTATGTGAACTCCCCAA  Reverse: 5′-AGTCCTATTGCCTGCCTGTT |
| Alanyl aminopeptidase | ANPEP | Forward: 5′-TGGCCACTACACAGATGCAG  Reverse: 5′-CTGGGACCTTTGGGAAGCAT |
| Kidney injury molecule-1 | KIM-1 | Forward: 5′- TCCGTGGCCCTTTTTGCTTA  Reverse: 5′- GGATCAGCGTTCAGATCCAGG |
| Glyceraldehyde-3-phosphate dehydrogenase | GAPDH | Forward: 5′-GTCTCCTCTGACTTCAACAG  Reverse: 5′-GTTGTCATACCAGGAAATGAG |
